# Hope, resilience and grit: a mediation model to predict burnout and psychological well-being

**DOI:** 10.1080/00049530.2026.2616975

**Published:** 2026-02-05

**Authors:** Mostafa Asheghi, Seyedmohammadjavad Mousavinia, Abdolzahra Naami

**Affiliations:** Industrial and Organizational Psychology, Faculty of Educational Sciences and Psychology, Shahid Chamran University of Ahvaz, Ahvaz, Iran

**Keywords:** Hope, resilience, grit, burnout, psychological well-being

## Abstract

**Objective:**

This study examined the relationships between hope, resilience, and grit with job burnout and psychological well-being, focusing on both direct effects and the mediating role of grit. Given the economic and organizational challenges in Iran, where employees frequently experience occupational stress, understanding these psychological resources is particularly important.

**Method:**

Participants were 300 employees from the Iran Oil Pipelines and Telecommunications Company in Lorestan, Iran. Five standardized questionnaires were administered, and data were analyzed using structural equation modeling (SEM) with SPSS 26 and AMOS 22.

**Results:**

The sample included 295 males and 5 females, with 66 single and 234 married individuals (Mean age = 40.98, SD = 10.28). SEM results showed that hope, resilience, and grit had significant direct effects on psychological well-being and burnout (β = -0.35–0.50, all p < .05). Grit also significantly mediated the relationships between hope and resilience with burnout and well-being.

**Conclusion:**

Hope, resilience, and grit are strong predictors of employees’ psychological well-being and burnout. In Iranian organizational contexts, strengthening these psychological resources through targeted interventions may help reduce occupational stress and enhance mental health.

## KEY POINTS

What is already known about this topic:

(1) Hope and resilience are well-established protective factors that reduce job burnout and enhance psychological well-being.

(2) Grit is recognised as a personality trait that supports sustained effort and persistence in challenging work environments.

(3) Previous research shows that personal resources like hope, resilience, and grit are linked to better occupational functioning and mental health outcomes.

What this topic adds:

(1) This study demonstrates the mediating role of grit in the relationship between hope, resilience, and employee outcomes in a high-stress Organisational environment.

(2) Provides empirical evidence on how psychological strengths interact to buffer against burnout and enhance well-being among Iranian employees.

(3) Offers practical insights for managers and practitioners to design interventions that cultivate hope, resilience, and grit in the workplace.

## Introduction

Employees in Iran’s industrial sector, particularly in the oil and petrochemical industries, face high levels of occupational stress due to demanding workloads, shift schedules, and safety-critical responsibilities (Askari et al., 2023; Hoboubi et al., 2017; Mokarami et al., 2021). Workers in these industries often operate under challenging conditions that may lead to job burnout and reduced psychological well-being. Job burnout has increasingly been recognised as a major global occupational health concern. A recent meta-analysis of public health workers, encompassing a total sample of 215,787 individuals across multiple countries, estimated the overall prevalence of burnout to be 39% (95% CI: 25–53%) (Nagarajan et al., 2024). In Iran, Bastami et al. (2020) reported high burnout rates across different occupational groups: 67% among librarians, 51% among university staff, and 72% among dentists. The World Health Organization (2024) has classified burnout as an “occupational phenomenon”, emphasising its widespread prevalence across diverse sectors. Burnout has been associated with substantial organisational and economic consequences, including reduced productivity, increased absenteeism, and higher turnover intentions. Given this global and national evidence – and considering that the present study focuses on employees of the Iran Petroleum Pipelines & Telecommunications Company, who operate in high-stress, safety-critical industrial roles – investigating psychological resources that may buffer burnout and support well-being is both timely and essential. Positive psychological resources, including hope, resilience, and grit, are important for coping with workplace stress and maintaining adaptive functioning. hope is defined as the determination to achieve goals and the belief that alternative pathways exist to reach them (Hefferon & Boniwell, 2011). Conceptually related to optimism, hope reflects an individual’s perceived capability to generate multiple pathways towards desired goals despite obstacles, as well as the motivational drive to pursue those pathways (Snyder, 2000). Another key personality trait influencing occupational and psychological outcomes is resilience. This capacity, which varies among individuals, allows employees to withstand and recover from workplace stressors, navigate challenging or hazardous environments, and adapt effectively to adverse experiences (Grotberg, 2003). Similarly, grit represents an important personal resource in the workplace. Beyond mere perseverance, grit reflects a sustained and often unseen form of determination that enables individuals to persist through obstacles, continuously strive for improvement in specific domains, and maintain long-term commitment to their goals (Lewis, 2014). Duckworth et al. (2007) examined the influence of passion and perseverance for long-term goals – collectively known as grit – to understand why some individuals of similar ability levels achieve greater success than others. Their findings highlighted grit as a key predictor of achievement across multiple domains of performance. It is proposed that these dispositional tendencies are more crucial than intellectual abilities in predicting long-term success. Generally, individuals with high levels of grit demonstrate the capacity to pursue personally meaningful goals over extended periods – weeks, months, or even years – despite facing obstacles, failures, or temporary setbacks (Duckworth et al., 2007). Conversely, individuals with lower grit tend to abandon or redirect their efforts towards alternative goals when confronted with similar barriers or lack of progress (Duckworth & Gross, 2014).

Considering the link between hope and grit, it has been proposed that counsellors and practitioners may foster individuals’ sense of hope, which in turn can enhance their levels of grit. Georgoulas-Sherr and Kelly (2019), using structural equation modelling to examine the interrelations among resilience, grit, and stubbornness, reported a positive association between resilience and grit. Both grit and resilience – concepts broadly referring to the capacity to persevere through challenges to achieve goals – have gained attention in both popular discourse and academic literature (Stoffel & Cain, 2018). These constructs are frequently highlighted as critical factors in coping with psychological stressors (Waxman et al., 2003). While grit and resilience are sometimes used interchangeably, they represent distinct constructs. Specifically, grit is characterised by sustained passion and persistence towards long-term objectives, reflecting a continuous commitment to pursue and complete tasks despite encountering failures, obstacles, or adversity (Duckworth et al., 2007).

To address the significant challenge of job burnout and psychological well-being among employees in high-stress Iranian industries, this study focused on three key psychological resources: hope, resilience, and grit. Hope, defined as the motivation and perceived ability to pursue goals despite obstacles (Hefferon & Boniwell, 2011; Snyder, 2000), has been shown to reduce burnout (Luthans et al., 2007; Reichard et al., 2013; Youssef & Luthans, 2007) and enhance well-being (Dursun, 2012; Kardas et al., 2019). Resilience, the capacity to adapt and recover from adversity (Grotberg, 2003), has similarly been shown to reduce burnout (Asheghi & Hashemi, 2019; Beddoe et al., 2013; Katsiroumpa et al., 2023; Lee et al., 2019; Nantsupawat et al., 2024; Strolin-Goltzman et al., 2016) and improve well-being in organisational environments (Andales et al., 2025; He et al., 2018; Ríos-Risquez et al., 2018; Lee & Hasson, 2020). Grit, representing sustained passion and perseverance towards long-term goals (Duckworth et al., 2007; Lewis, 2014), not only strengthens the effects of hope and resilience but can also directly influence burnout and well-being. By examining both the direct effects and the indirect effects mediated by grit, this study aims to clarify how these psychological resources operate in reducing burnout and promoting well-being, addressing gaps in prior research that have largely overlooked their combined role in occupational environments.

## Hope and employee outcomes

According to Snyder’s Hope Theory (Snyder, 1994), hope consists of two components: agency, the determination to achieve goals, and pathways, the perceived ability to find ways to reach them. Employees with higher levels of hope are more likely to stay motivated, set clear goals, and overcome obstacles at work. Hope functions as a psychological resource that enhances well-being and protects employees from burnout. Research shows that hope positively relates to job satisfaction and commitment, while reducing stress and exhaustion (Luthans et al., 2007; Reichard et al., 2013; Youssef & Luthans, 2007). In demanding work environments – like those in many Iranian organisations with limited resources and high workload – hope helps employees maintain purpose and emotional stability. Thus, hopeful employees tend to experience less burnout and greater psychological well-being. Research also indicates that hope can contribute to enhanced psychological well-being (Dursun, 2012; Kardas et al., 2019). Essentially, hope refers to having the motivation to pursue one’s goals and the ability to plan effectively to achieve them. From this perspective, goal-directed motivation and planning are expected to play a significant role in improving an individual’s quality of life and, consequently, enhancing well-being. Therefore, hope is a key variable closely linked to psychological well-being (Kardas et al., 2019) and has a negative relationship with burnout (Yavas et al., 2013).

It appears that hope plays a significant role in guiding an individual’s cognitive processes and behaviours. Due to the general tendency of hopeful individuals to repeatedly experience positive mood states and goal-oriented positive outlooks, hopeful employees may be less susceptible to job burnout. On the other hand, because of their inherent tendency to find ways to overcome challenges, they may perform effectively even in the face of burnout. As the broad and constructive effects of hope accumulate and interact over time, higher levels of hope can foster positive change, making individuals more resilient and effective (Yavas et al., 2013).

## Resilience and employee outcomes

Resilience has been defined in multiple ways (Cassidy, 2015), yet it is generally understood as an individual’s capacity to maintain or restore psychological well-being following exposure to adversity (Herrman et al., 2011). It represents a dynamic process through which individuals adapt positively to challenging or adverse experiences (Masten, 2001). Frequently, resilience is also described using terms such as “stress resistance” (Garmezy, 1985) or “post-traumatic growth” (Tedeschi et al., 1998), reflecting the spectrum of responses individuals exhibit when confronted with psychological trauma. In this context, resilience is recognised not only as an outcome but also as a cognitive process that facilitates adaptation and recovery (Ingram & Price, 2001). According to the American Psychological Association (2020), resilience refers to the dynamic process through which individuals effectively adjust to challenging circumstances such as trauma, threats, or major stressors. Similarly, Lee and Cranford (2008) describe it as the ability to successfully manage substantial life changes, obstacles, or risks, while Connor and Davidson (2003) consider it a personal resource that allows individuals to maintain growth and functioning in the face of hardship. Empirical studies further show that resilience contributes to reducing employee burnout (Asheghi & Hashemi, 2019; Beddoe et al., 2013; Katsiroumpa et al., 2023; Lee et al., 2019; Nantsupawat et al., 2024; Strolin-Goltzman et al., 2016). and improving well-being in Organisational environment (He et al., 2018; Ríos-Risquez et al., 2018, Lee & Hasson, 2020; Andales et al., 2025).

burnout arises from prolonged exposure to occupational stress and is defined as a syndrome characterised by emotional exhaustion, depersonalisation, and a diminished sense of personal accomplishment (Schaufeli et al., 2017). It represents a chronic emotional condition often accompanied by fatigue, psychological depletion, and cognitive exhaustion. Employees experiencing burnout typically exhibit reduced motivation and impaired professional efficacy. However, resilient individuals tend to recover more rapidly from such conditions, maintaining psychological equilibrium and better emotional and physical health despite demanding circumstances (Jaureguizar et al., 2018).

Under such circumstances, resilient individuals experience lower levels of burnout. Moreover, resilience has been shown to enhance psychological well-being (He et al., 2018; Ríos-Risquez et al., 2018; Lee & Hasson, 2020). Fredrickson (2001) suggested that resilience plays a crucial role in promoting psychological well-being by enabling individuals to build and sustain positive emotional resources. Similarly, Ryff and Singer (2003) argued that resilient individuals are generally better able to preserve their physical and mental health and recover more rapidly from stressful life events. Consequently, resilience contributes to greater overall psychological well-being.

## Grit as a mediator

Theorists conceptualise grit as a higher-order construct composed of two interrelated dimensions: consistency of interest and persistence of effort (Duckworth et al., 2007). Duckworth (2016) compares consistency of interest to a compass – something developed and refined over time that provides direction and sustains an individual’s commitment to their long-term aspirations. In this sense, consistency of interest reflects one’s enduring capacity to maintain focus and enthusiasm for long-term goals. Persistence of effort, on the other hand, represents the sustained energy and determination to continue working towards objectives despite challenges and discouragement (Duckworth & Gross, 2014). Individuals high in grit remain steadfast in pursuing their overarching goals over time, setting aside conflicting ambitions and revising subordinate goals when necessary to stay aligned with their ultimate purpose (Duckworth & Gross, 2014).

Empirical evidence suggests that grit is associated with multiple indicators of life quality (Jin & Kim, 2017). Individuals with higher levels of grit tend to report greater positive affect (Hill et al., 2014), enhanced hope and optimism (Sheridan et al., 2015; Vela et al., 2018), as well as higher levels of psychological well-being, life satisfaction, and overall harmony in life (Vainio & Daukantaitė, 2016). Conversely, grit demonstrates a negative association with burnout (Al-Zain & Abdulsalam, 2022; Brateanu et al., 2020; Dam et al., 2019; Salles et al., 2014), anxiety (Sheridan et al., 2015), and symptoms of depression or suicidal ideation (Kleiman et al., 2013). Moreover, existing research highlights significant interconnections between grit, hope, and resilience. For example, Vela et al. (2018) examined the predictive role of positive psychological factors in developing grit among Latino and college students, revealing that both hope and mindfulness serve as key predictors of psychological grit. When individuals believe in their capacity to build a positive future, their enthusiasm and perseverance towards long-term goals tend to strengthen. Similarly, prior studies have shown that hope contributes to goal attainment (Feldman et al., 2009) and supports better mental health outcomes (Marques et al., 2011).

Given its motivational nature, grit may serve as a mediating mechanism through which hope and resilience influence employee outcomes. Specifically, employees with higher hope and resilience tend to sustain their effort towards goals, even in adverse conditions, due to their grittier approach to work. Consequently, grit channels the positive effects of hope and resilience towards lower burnout and higher well-being, offering a comprehensive explanation for the observed relationships in organisational workplaces.

**Figure 1.**
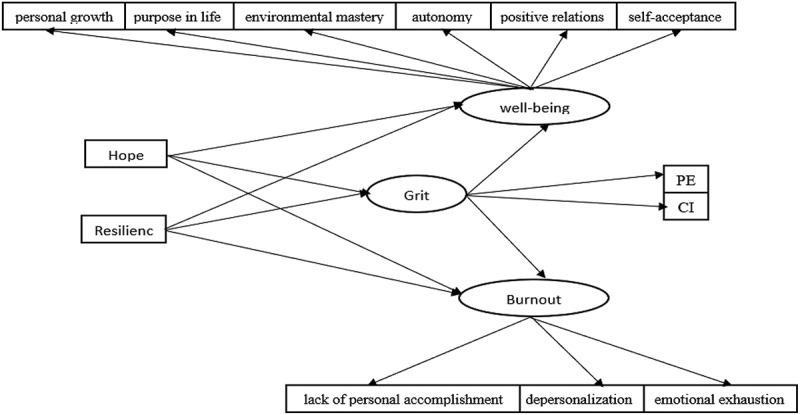
The hypothetical model of the mediation role of grit.

Moreover, resilience is considered a core component of grit. Given the limited empirical research on grit, the present study aims to investigate the relationships between hope, resilience, and key organisational outcomes, specifically job burnout and psychological well-being, while examining the mediating role of grit. To this end, a conceptual model (Figure 1) was proposed and tested. Accordingly, the central research question of this study is: To what extent does the proposed model demonstrate an acceptable fit?

## Methods

### Study design and participants

This study employed a correlational research design with structural equation modelling (SEM) to examine the relationships among hope, resilience, grit, job burnout, and psychological well-being. The statistical population consisted of all employees of the Iran Petroleum Pipelines and Telecommunications Company in the Lorestan region in 2025. A total of 300 participants were selected using a convenience sampling method to test the proposed research model. Following Klein’s recommendation that at least 10 participants are required per estimated parameter (Bashlide, 2014), the study calculated 14 parameters based on the number of direct paths (8), exogenous variables (2), covariances (1), and error variances (3). With 300 participants, approximately 21 participants were allocated per parameter, indicating that the sample size was sufficient for SEM analysis.

### Ethical considerations

Ethical approval for this study was obtained from Shahid Chamran University of Ahvaz. All participants provided informed consent prior to participation. They were informed about the study’s aims, the voluntary nature of participation, and their right to withdraw at any time without penalty. Data were collected via structured questionnaires during working hours. To ensure anonymity, no identifying information (e.g., names, personnel codes, or contact details) was collected, and responses were analysed and reported only in aggregated form. Confidentiality of the data was strictly maintained throughout all stages of the study.

## Measures

### Hope scale

In the present study, the hope subscale of the Psychological Capital Questionnaire was employed to assess participants’ level of hope (Luthans et al., 2007). This subscale comprises six items, with respondents rating each item on a six-point Likert scale (Luthans et al., 2007). The construct validity of the scale was supported through confirmatory factor analysis, with fit indices indicating an acceptable model fit: relative chi-square (χ^2^/df = 2.31), Incremental Fit Index (IFI = 0.90), Comparative Fit Index (CFI = 0.90), and Root Mean Square Error of Approximation (RMSEA = 0.06). Reliability analyses further demonstrated that the scale had satisfactory internal consistency, with a Cronbach’s alpha of 0.85 reported in previous research, and 0.91 in the current study, confirming the scale’s reliability.

### Resilience scale

Resilience questionnaire (Connor & Davidson, 2003) is a 25-item instrument used to measure resilience. Its items are rated on a 5-point Likert scale (0 for “Totally incorrect” and a score of 4 for “Absolutely right”). the reliability of this questionnaire was obtained through Cronbach’s alpha of 0.93, which indicates the reliability of this questionnaire (Mohammadi, 2005). In this study, Cronbach’s alpha coefficient was 0.96, confirming the reliability of the scale.

### Grit scale

The Grit Questionnaire (Duckworth & Quinn, 2009) is a 12-item instrument used to measure grit. The grit questionnaire consists of two 4-item subscales that measure persistence in effort (PE) and consistency of interest (CI), respectively. In their meta-analysis of grit at different ages, Credé et al. (2017) showed the average Cronbach’s alpha coefficient for the long form and short form of the grit scale to be 0.79. The average Cronbach’s alpha coefficient for the persistence in effort subscale was 0.71 and for the consistency of interest subscale was 0.74. In this study, Cronbach’s alpha coefficient was 0.95, confirming the reliability of the scale.

### Burnout scale

Burnout Questionnaire (Maslach & Jackson, 1981) is 22 items, that 9 items are related to emotional exhaustion, 8 items related to the lack of personal accomplishment and 5 items related to depersonalisation. Maslach and Jackson (1984) reported good internal consistency (alpha .83) for the scale. In this study, Cronbach’s alpha coefficient was 0.96, confirming the reliability of the scale.

### Well-being scale

Well-Being Scale (Ryff’s, 1989) is 25 items, this instrument consists of 18 items across six dimensions – self-acceptance, positive relations with others, autonomy, environmental mastery, purpose in life, and personal growth. Its items are rated on a 6-point Likert scale (0 for “strongly agree” and a score of 6 for “strongly disagree”). In the original American sample, Ryff reported test-retest reliability coefficients between 0.81 and 0.86, and internal consistency values for the subscales ranging from 0.86 to 0.93. the reliability of this questionnaire was obtained through Cronbach’s alpha of 0.82, which indicates the reliability of this questionnaire (Bayani et al., 2008). In this study, Cronbach’s alpha coefficient was 0.95, confirming the reliability of the scale.

### Data analysis

The conceptual framework illustrated in Figure 1 was evaluated using structural equation modelling (SEM) in AMOS 24. To assess the model’s goodness-of-fit, a combination of indices was employed, including the relative chi-square (χ^2^/df), Incremental Fit Index (IFI), Comparative Fit Index (CFI), Goodness-of-Fit Index (GFI), Adjusted Goodness-of-Fit Index (AGFI), Tucker-Lewis Index (TLI), and the Normed Fit Index (NFI) (Bentler & Bonett, 1980). Table 1 presents descriptive statistics and correlation coefficients for the study variables. As indicated, all correlation coefficients were statistically significant at the *p* < 0.05 level.

**Table 1.** Descriptive statistics and bivariate-correlations for research variables.

Variables		M	SD	1	2	3	4	5
1	hope	24.180	8.754	–				
2	resilience	83.046	26.700	.70**	–			
3	grit	40.366	13.776	.50**	.60**	–		
4	psychological well-being	70.533	22.946	.66**	.73**	.72**	–	
5	burnout	46.793	32.777	−.71**	−.72**	−.66**	−.84**	–

*Note*. **Correlation is significant at the 0.01 level (2-tailed).

## Results

**Figure 2.**
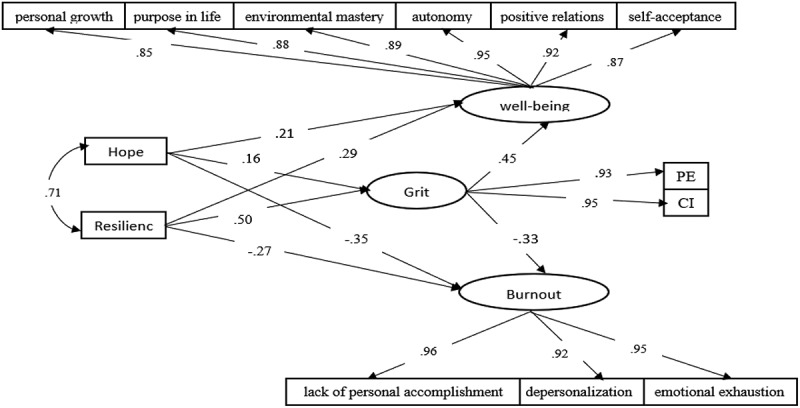
Standardized regression weights for the final model paths.

Note: **p < .001.

The demographic characteristics of the participants included 295 men and 5 women, 66 single and 234 married individuals, (Mean age = 40.98, SD = 10.28). Regarding educational background, participants were distributed as follows: Diploma (n = 32), Associate degree (n = 51), Bachelor’s degree (n = 159), Master’s degree (n = 54), and PhD (n = 8). An Independent Samples t-test was conducted to examine potential gender differences in job burnout. Levene’s test indicated that the assumption of homogeneity of variances was met, F = 0.857, *p* = 0.355. No significant differences were found between male (M = 46.57, SD = 32.68) and female participants (M = 59.60, SD = 39.83), t (298) = −0.881, *p* = 0.379, Mean Difference = −13.02, 95% CI [−42.12, 16.07]. These results suggest that gender did not significantly influence burnout levels in this sample. Mean burnout scores across education levels were as follows: Diploma: M = 57.10, SD = 35.86; Associate degree: M = 41.43, SD = 26.83; Bachelor: M = 45.50, SD = 32.22; Master: M = 53.70, SD = 37.35; PhD: M = 23.75, SD = 11.84. A one-way ANOVA indicated a significant effect of education on burnout, F (4, 295) = 2.74, *p *= .029. However, post-hoc comparisons using the Tukey HSD test did not reveal any statistically significant pairwise differences between the education levels (all *p* > .05). These results suggest that while education level may have a general influence on burnout, no specific group differed significantly from another, indicating that other factors may contribute more prominently to burnout in this sample. Mean well-being scores across education levels were as follows: Diploma: M = 61.17, SD = 24.45; Associate degree: M = 71.82, SD = 20.78; Bachelor: M = 72.49, SD = 21.93; Master: M = 65.87, SD = 26.08; PhD: M = 87.50, SD = 11.73. A one-way ANOVA indicated a significant effect of education on well-being, F (4, 295) = 3.24, *p* = .013. Post-hoc comparisons using Tukey’s HSD revealed that participants with a PhD reported significantly higher well-being than those with a Diploma (*p* = .032). No other pairwise comparisons reached statistical significance. Means, standard deviations, and the bivariate correlations among the variables are presented in Table 1. As presented in Table 2, the fit indices following model modification indicate that the proposed conceptual model demonstrates a satisfactory fit. To enhance model fit, error covariances were added between variables exhibiting high correlations, specifically linking psychological well-being and job burnout. The resulting goodness-of-fit indices were as follows: relative chi-square (χ^2^/df = 2.68), Incremental Fit Index (IFI = 0.99), Comparative Fit Index (CFI = 0.98), Goodness-of-Fit Index (GFI = 0.97), Adjusted Goodness-of-Fit Index (AGFI = 0.92), Tucker-Lewis Index (TLI = 0.98), Normed Fit Index (NFI = 0.98), and Root Mean Square Error of Approximation (RMSEA = 0.07). These results collectively indicate that the hypothesised model fits the observed data well. Bootstrapping procedures were used to test the mediation paths. We generated 5000 bootstrapping samples from the original dataset (N = 300). Table 3 displays the direct effects and Table 4 exhibits the indirect effects and their associated 95% confidence intervals. As shown in Table 4, the upper and lower limits of the confidence intervals for all two indirect paths do not include zero, indicating that all four indirect paths are statistically significant. Therefore, grit serves as a mediator in the relationship between hope and resilience with both psychological well-being and burnout. Figure 2 presents the final model and standardised regression weights.

**Table 2.** Fit indices among competing models.

Variable fit indices	χ^2^	df	$\chi^{2}/\mathrm{df}$	AGFI	GFI	NFI	CFI	IFI	TLI	RMSEA
Acceptable values	–	–	<5	≥.90	≥.90	≥.90	≥.90	≥.90	≥.90	≤.080
Proposed model	34.89	13	2.68	.92	.97	.98	.99	.99	.98	.07

*Note*. *N* = 300; RMSEA = Root Mean Square Error of Approximation; CFI=Comparative Fit Index; IFI= Incremental Fit Index; and NFI=Normed Fit Index.

**Table 3.** Direct effects for the final mediational model.

Paths	Standard Estimates	Non-standard estimation	S.E.	C.R.	P Label
hope → grit	.16	.12	.05	2.49	.013
hope → psychological well-being	.21	.57	.12	4.70	.001
hope → burnout	−.35	−.53	.07	−7.29	.001
Resilience → grit	.50	.12	.01	7.47	.001
Resilience → psychological well-being	.29	.25	.04	5.70	.001
Resilience → burnout	−.27	−.13	.02	−5.14	.001
grit → psychological well-being	.45	.89	.15	10.12	.001
grit → burnout	−.33	−.66	.09	−7.22	.001

**Table 4.** Indirect effects for the final mediational model.

Indirect paths	Indirect effect	95% confidence interval		*P*
		Lower	Upper	
hope → grit → psychological well-being	.076	.027	.130	.011
hope→ grit → burnout	−.057	−.099	−.022	.014
Resilience → grit → psychological well-being	.231	.176	.297	.000
Resilience → grit → burnout	−.172	−.232	−.125	.000

## Discussion

The present study aimed to investigate the relationships between hope and resilience with job burnout and psychological well-being, with grit considered as a mediating factor. The results indicated that both hope and resilience had significant direct positive effects on psychological well-being and negative effects on burnout. Moreover, grit served as an important mediator, transmitting and amplifying the positive influence of hope and resilience on well-being while buffering against burnout. All path coefficients for both direct and indirect relationships were statistically significant, highlighting the critical role of personal psychological resources in organisational environments. Although this study focused on industrial employees in Iran, job burnout and psychological distress are not confined to industrial sectors. Previous research in healthcare has demonstrated similar challenges. A study on critical care nurses found that high levels of anxiety, depression, and stress were significantly associated with increased job burnout and poor sleep quality (Gravante et al., 2025). Similarly, a systematic review on moral injury in nurses reported that moral distress was linked to elevated anxiety, depression, and reduced quality of life (Anastasi et al., 2025). These findings indicate that job-related stress and burnout are widespread across professions, underscoring the need to examine protective psychological resources such as hope, resilience, and grit in diverse occupational environments. Moreover, education level may influence how individuals cope with burnout, with higher educational attainment generally associated with more effective strategies, although no specific group differences were significant in our sample.

### Association of hope with grit, psychological well-being, burnout

According to Table 3, results showed that hope had a positive effect on grit (β = .16, *p* = .013). Hope, as a positive psychological construct, reflects an individual’s capacity to identify alternative pathways towards desired goals and the motivational drive to pursue them (Snyder, 2000). Since grit encompasses sustained effort and perseverance in achieving long-term objectives (Duckworth et al., 2007), individuals with higher levels of hope are likely to demonstrate greater commitment and persistence when facing obstacles. Empirical studies have shown that hope can serve as a predictor of grit, as hopeful individuals are generally more resilient and dedicated in pursuing their goals (Vela et al., 2018). Therefore, it is expected that hope will be positively associated with grit.

The results indicate that hope positively influences psychological well-being (β = .21, *p* = .001) (Dursun, 2012; Kardas et al., 2019). Conceptually, hope reflects both the drive to pursue important goals and the capacity to plan strategies to achieve them. Individuals with higher levels of hope are more likely to perceive challenges as manageable and to identify multiple strategies for achieving their objectives. This adaptive mindset promotes resilience, positive emotions, and engagement with meaningful activities, which are core components of psychological well-being (Ryff, 1989). Empirical evidence indicates that hopeful individuals report higher levels of life satisfaction, autonomy, personal growth, and positive relationships (Dursun, 2012; Kardas et al., 2019). Additionally, hope had a negative effect on burnout (β = −.35, *p* = .001). Empirical evidence consistently indicates an inverse relationship between hope and job burnout (Dursun, 2012; Yavas et al., 2013).

Hope appears to play a critical role in shaping cognitive processes and guiding behaviour. Employees with higher levels of hope may be less susceptible to burnout, as they tend to maintain positive moods and adopt goal-oriented perspectives even in challenging circumstances. Additionally, their intrinsic drive to identify solutions and overcome obstacles enables them to sustain performance despite fatigue or stress.

### Association of resilience with grit, psychological well-being, burnout

In the present study, results showed that resilience had a positive effect on grit (β = .50, *p* = .001). Resilience, a capacity present in certain individuals, allows people to navigate life’s difficulties and cope effectively with environmental adversities (Grotberg, 2003). Consequently, resilient employees often demonstrate higher levels of dedication and grit in the workplace (Georgoulas-Sherr & Kelly, 2019).

Resilience demonstrated a positive effect on psychological well-being (β = .29, *p* = .001). Ryff and Singer (2003) note that resilient individuals are generally able to maintain both physical and mental health and recover more rapidly from stressful events, which contributes to higher levels of psychological well-being. Resilience helps individuals maintain psychological balance and recover more quickly when facing life stressors and adversities. This ability enhances their sense of control, self-confidence, and life satisfaction. Consequently, resilient individuals typically exhibit higher levels of psychological well-being and are better able to pursue their personal and social goals effectively (Andales et al., 2025; He et al., 2018; Ríos-Risquez et al., 2018, Lee & Hasson, 2020).

Additionally, results showed that resilience had a negative effect on burnout (β = −.27, *p* = .001). Empirical evidence consistently indicates an inverse relationship between resilience and job burnout (Asheghi & Hashemi, 2019; Beddoe et al., 2013; Katsiroumpa et al., 2023; Lee et al., 2019; Nantsupawat et al., 2024; Strolin-Goltzman et al., 2016).

Resilient employees tend to restore emotional and physical balance more quickly following experiences of burnout, thereby experiencing reduced intensity and duration of burnout symptoms (Jaureguizar et al., 2018). resilience equips individuals with the psychological resources to effectively manage workplace stressors and adapt to challenging circumstances. Employees with higher resilience are less vulnerable to the key dimensions of job burnout – emotional exhaustion, depersonalisation, and reduced personal accomplishment. Consequently, resilience functions as a robust protective factor, buffering the adverse effects of occupational stress and lowering both the likelihood and severity of burnout.

### Influence of grit on psychological well-being and burnout

results showed that grit had a positive effect on psychological well-being (β = .45, *p* = .001) and mediated the relationship between hope and resilience with psychological well-being and burnout. The findings of this study can be theoretically grounded in Rogers’ Organismic Valuing Theory (Rogers, 1964), which emphasises the individual’s inherent tendency towards growth and self-actualisation. According to Rogers, psychological well-being arises when individuals pursue goals that are congruent with their authentic self and intrinsic values. In this sense, grit – defined as perseverance and passion for long-term goals – reflects the motivational consistency and inner drive that propel individuals towards self-realisation. Empirical studies have also demonstrated that individuals high in grit report greater life satisfaction, purpose, and emotional stability (Al-Zain & Abdulsalam, 2022; Andales et al., 2025; Duckworth et al., 2007; Bowman et al., 2015). Therefore, grit can be interpreted as a behavioural manifestation of the self-actualising tendency described by Rogers, serving as a psychological mechanism that supports sustained engagement with meaningful life pursuits and, consequently, enhances overall well-being (Vainio & Daukantaitė, 2016). Grit aligns closely with Rogers’ concept of the innate tendency towards growth and self-actualisation, as proposed in the Organismic Valuing Theory. Individuals high in grit consistently pursue ambitious and meaningful long-term goals that demand sustained self-regulation and a strong sense of authenticity. This congruence between perseverance, purpose, and the true self ensures that their continuous efforts contribute positively to psychological well-being and personal fulfilment.

The results of Table 3 revealed that grit was directly and negatively associated with burnout (β = −.33, *p* = .001). Empirical evidence consistently indicates an inverse relationship grit and burnout (Al-Zain & Abdulsalam, 2022; Brateanu et al., 2020; Dam et al., 2019; Salles et al., 2014). Individuals with higher levels of grit possess greater persistence in effort (PE) and consistency of interest on long-term goals, which enables them to cope more effectively with workplace challenges and setbacks. Grit, comprising consistency of interest and persistence of effort, allows individuals to maintain their motivation and engagement even under stressful conditions, thereby preventing premature disengagement or withdrawal.

### Mediating role of grit

According to Table 4, the results of the study showed that grit has a mediating role in the relationship between hope and resilience with psychological well-being and burnout. Individuals with higher hope and resilience are more likely to develop grit, characterised by greater persistence in effort (PE) and consistency of interest towards long-term goals. This persistent engagement enhances psychological well-being by fostering self-regulation, goal-directed behaviour, and a sense of personal growth, while simultaneously mitigating burnout by reducing emotional exhaustion, depersonalisation, and the lack of personal accomplishment. These findings align with Rogers’ organismic valuing theory, which posits that self-motivated individuals pursue authentic goals that promote personal fulfilment and long-term growth. Consequently, grit functions as a protective and facilitative factor, translating positive psychological resources such as hope and resilience into improved workplace outcomes.

### Limitations and future research

This study aimed to examine the roles of hope and resilience in predicting burnout and psychological well-being, with grit as a mediating factor. However, several limitations should be acknowledged when interpreting the findings. First, the cross-sectional design restricts causal inferences; thus, longitudinal studies are recommended to explore the causal relationships among these variables. Second, the sample was drawn from employees of a single organisation in a specific region, which may limit the generalisability of the results to other organisations or industries. Additionally, demographic heterogeneity regarding gender and other characteristics could have influenced the observed relationships. Based on the findings, it is suggested that organisations implement training programmes and interventions aimed at enhancing employees’ hope, resilience, and grit. Such interventions may reduce burnout and promote psychological well-being. Future research could also investigate the roles of other potential mediators and moderators, such as social support, emotional intelligence, and stress management skills, to provide a more comprehensive understanding of the psychological mechanisms underlying burnout prevention and well-being enhancement.

## Conclusion

This study highlights the significance of positive psychological resources – namely hope, resilience, and grit – in high-pressure organisational environments. The findings indicate that employees who possess higher levels of these resources are better equipped to pursue meaningful goals, maintain psychological well-being, and cope effectively with workplace stressors. From a practical standpoint, interventions aimed at strengthening these personal capacities may serve as an effective approach to reducing burnout and enhancing mental health and productivity within organisations. Overall, by demonstrating both the direct effects and the indirect (grit-mediated) pathways through which hope and resilience influence burnout and well-being, this study contributes to the existing literature and addresses a notable gap – specifically, the lack of attention to the combined role of multiple individual psychological resources in occupational settings. Future research is encouraged to examine these relationships across different professions, industries, and cultural contexts to better establish the generalisability of the findings.

## Data Availability

Data sharing is not available for this article due to the sensitive nature of the research.
